# Case Series of Monoamniotic and Pseudomonoamniotic Twin Gestations

**DOI:** 10.1155/2013/369419

**Published:** 2013-02-21

**Authors:** Shunji Suzuki

**Affiliations:** Department of Obstetrics and Gynecology, Japanese Red Cross Katsushika Maternity Hospital, 5-11-12 Tateishi, Katsushika-ku, Tokyo 124-0012, Japan

## Abstract

We retrospectively evaluated a series of 18 monoamniotic and 7 pseudomonoamniotic (secondary to rupture in the membrane dividing monochorionic diamniotic twins) twin gestations managed after 20 weeks' gestation. There were no significant differences in the incidence of neonatal death or umbilical cord entanglement between the monoamniotic and pseudomonoamniotic twin gestations (33 versus 21%, *P* = 0.94 and 72 versus 43%, *P* = 0.36). Therefore, the same serious management may be needed for pseudomonoamniotic twin gestations as for monoamniotic twin gestations.

## 1. Introduction

Monoamniotic twinning is a rare complication, occurring in less than 1% of monozygosity and is associated with a significant mortality rate [1, 2]. The most common cause of perinatal mortality in monoamniotic twins has been reported to be cord entanglement [1, 2]. Cord entanglement has been reported in up to 70% of monoamniotic twins with 50% or more of deaths attributed to this complication [3]. Recently, some cases of disruption of the dividing membrane in monochorionic diamniotic twin gestation as pseudomonoamniotic twin gestation [4–13]. In some previous reports, pseudomonoamniotic twin gestation sometimes has been also observed to be complicated by umbilical cord entanglement. In addition, some cases of monochorionic diamniotic twin pregnancy complicated by spontaneous antepartum rupture of the intertwin-dividing membrane (and umbilical cord entanglement) without any perinatal episodes have been reported [12, 13]; however, there have been few investigations comparing the perinatal outcomes in pseudomonoamniotic twins with those in true monoamniotic twins. Therefore, in this study, we examined the perinatal outcomes of these pregnancies.

## 2. Methods

We retrospectively evaluated a series of 18 monoamniotic and 7 pseudomonoamniotic twin gestations managed after 20 weeks' gestation at our hospital (these contain the cases we reported previously [6–8, 14]). The diagnosis of monoamnionicity and pseudomonoamnionicity was determined on the basis of ultrasound features and it was confirmed by clinical presentations at delivery, such as the presence of disruption of the dividing membrane and histological examination of the placenta. The histology of the placenta and umbilical cord entanglement were examined at the time of delivery. Cases of congenital anomalies such as anencephaly and acardiac twins were excluded. In addition, the pregnancies were excluded, if fetal demise at least one twin at <20 weeks' gestation was diagnosed. The gestational age of the pregnancies was established by ultrasonographic examination of the fetal crown-rump length at 8–11 weeks' gestation.

In our hospitals, elective cesarean at preterm for (true and pseudo-) monoamniotic twin gestations as previously reported [15, 16] was not performed without maternal request, if fetuses were well and the patient had no maternal complications. In monoamniotic twin gestations, twin-twin transfusion syndrome (TTTS) was diagnosed with the clinical presentations, such as cardiac dysfunction, discordance in bladder size, and/or polyhydramnios.

Cases and controls were compared by the *χ* ^2^ or Fisher's exact test for categorical variables. Differences with *P* < 0.05 were considered significant.

## 3. Results

Table 1 shows the summary of perinatal findings in the 18 (true) monoamniotic twin gestations. The incidence of perinatal loss per total number of neonates was 33% (12/36). Four cases (22%) resulted in double death, while 4 (22%) cases resulted in single death. The incidence of cerebral injury per total number of lived neonates was 13% (3/24). The incidence of umbilical cord entanglement was 72% (13/18). There were no cases of severe congenital heart anomalies. Based on the clinical presentations, 2 cases (11%: cases 4 and 9) were suggested to be complicated by TTTS.

Table 2 shows the summary of perinatal findings in the 7 pseudomonoamniotic twin gestations. In 3 cases of these (43%), the disruption of the dividing membrane was associated with the therapies (amniocentesis and fetoscopic laser photocoagulation) for TTTS. As our impression, at the deliveries the area of the perforated portions of dividing membranes seemed to be large enough to lead to umbilical cord entanglement. Of 4 cases with spontaneous membrane rupture, 2 mothers (cases 5 and 7) felt the amniotic fluid flow due to membrane rupture of the second twin before labor. The incidence of perinatal loss per total number of neonates was 21% (3/14). One case (14%) resulted in double death, while 1 (14%) case resulted in single death during the second trimester. The incidence of umbilical cord entanglement was 43% (3/7). There were no cases of cerebral injury or severe congenital heart anomalies.

There were no significant differences in the incidence of neonatal death or umbilical cord entanglement between the monoamniotic and pseudomonoamniotic twin gestations (*P* = 0.94 and 0.36). Thirteen of 15 perinatal losses (87%) were occurred before 32 weeks of gestation.

## 4. Discussion

To date, some possible mechanisms leading to antepartum rupture of the intertwin-dividing membrane except artificial septostomy, such as amniocentesis and fetoscopic laser photocoagulation, have been proposed, such as infection (chorioamnionitis) [9], developmental disturbance [9], trauma or physical rupture by fetuses [10], and intrauterine sling formation [11]. In addition, some cases of spontaneous antepartum rupture, in which the exact cause of rupture of membrane cannot be well determined, have been reported. In our 7 cases of pseudomonoamniotic twin gestations, 3 cases were artificial and 4 cases were spontaneous.

 We know that the sample size of this study is very small; however, the current results may indicate that the perinatal outcomes of pseudomonoamniotic twin gestations do not differ from those of true monoamniotic twin gestations. Based on the results, the incidence of umbilical cord entanglement, which is the most critical concern in twin gestations, in monoamniotic twins seemed to be similar to that in pseudomonoamniotic twins. As our impression, the area of the perforated portions of dividing membranes seemed to be large enough to lead to umbilical cord entanglement. In addition, there was no significant difference in perinatal loss between the monoamniotic and pseudomonoamniotic twins. Therefore, the same serious management may be needed for pseudomonoamniotic twin gestations as for monoamniotic twin gestations.

 In this study, the total incidence of perinatal death in monoamniotic and pseudomonoamniotic twin gestations seemed to be high as previously reported [1, 2]; however, 87% of perinatal losses were occurred before 32 weeks of gestation, and there was no occurrence of new perinatal deaths after 34 weeks of gestation. In some literatures, the recommended timing of delivery range to prevent sudden intrauterine fetal death has been reported to be between 32 and 34 weeks [1, 15, 16]. However, the current result may support the other articles that the incidence of fetal death after 32 weeks is not high in monoamniotic twins [17, 18], which is suggesting that prophylactic preterm delivery may not be indicated in all monoamniotic twin gestations. Therefore, a further large study may be needed concerning the appropriate timing of delivery for monoamniotic and pseudomonoamniotic twins.

## 5. Conclusion

The same management is needed for monoamniotic and pseudomonoamniotic twin gestations.

## Figures and Tables

**Table 1:** Summary of perinatal findings in 18 monoamniotic twin gestations.

Number	GA at delivery	Twin A	Twin B	UC entanglement	Other complications
1	20	IUFD	IUFD	Yes	
2	22	IUFD	IUFD	Yes	
3	28	CP	IUFD	Yes	
4	28	Neonatal death	IUFD (22 weeks)	No	Oligohydramnios following polyhydramnios
5	29	CP	Healthy	Yes	Hydrops
6	30	IUFD (26 weeks)	Healthy	Yes	
7	31	Healthy	Healthy	Yes	
8	32	Healthy	Healthy	No	
9	32	IUFD (28 weeks)	PVL	No	Hydrops (IUFD), polyhydramnios
10	33	IUFD	IUFD	Yes	
11	34	Healthy	Healthy	Yes	
12	35	Healthy	Healthy	Yes	
13	36	Healthy	Healthy	Yes	
14	36	Healthy	Healthy	Yes	
15	37	Healthy	Healthy	Yes	
16	37	Healthy	Healthy	No	
17	38	Healthy	Healthy	Yes	
18	39	Healthy	IUFD (20 weeks)	No	

GA: gestational age; UC: umbilical cord; IUFD: intrauterine fetal death; CP: cerebral palsy; PVL: periventricular leukomalacia.

**Table 2:** Summary of perinatal findings in 7 pseudomonoamniotic twin gestations.

Number	GA at delivery	Twin A	Twin B	UC entanglement	Cause of membrane rupture
1	27	IUFD	IUFD	No	Amnioreduction due to TTTS
2	28	Healthy	IUFD (21 weeks)	Yes	Single IUFD
3	31	Healthy	Healthy	No	FLP due to TTTS
4	33	Healthy	Healthy	No	FLP due to TTTS
5	35	Healthy	Healthy	No	Unknown
6	36	Healthy	Healthy	Yes	Unknown
7	37	Healthy	Healthy	Yes	Unknown

GA: gestational age; UC: umbilical cord; IUFD: intrauterine fetal death; TTTS: twin-twin transfusion syndrome; FLP: fetoscopic laser photocoagulation.

## References

[1] Hack KE, Derks JB, Schaap AH (2009). Perinatal outcome of monoamniotic twin pregnancies. *Obstetrics and Gynecology*.

[2] Dickinson JE (2005). Monoamniotic twin pregnancy: a review of contemporary practice. *Australian and New Zealand Journal of Obstetrics and Gynaecology*.

[3] Su LL (2002). Monoamniotic twins: diagnosis and management. *Acta Obstetricia et Gynecologica Scandinavica*.

[4] Aisenbrey GA, Catanzarite VA, Hurley TJ, Spiegel JH, Schrimmer DB, Mendoza A (1995). Monoamniotic and pseudomonoamniotic twins: sonographic diagnosis, detection of cord entanglement, and obstetric management. *Obstetrics and Gynecology*.

[5] Lee KJ, Kim MK, Lee SY, Lee WS, Lee YH (2012). Spontaneous rupture of thedividing membrane in a monochorionic pregnancy resulting in a pseudo-monoamniotic pregnancy with cord entanglement. *Journal of Obstetrics and Gynaecology Research*.

[6] Miura N, Suzuki S (2008). Fetal asphyxia due to cord entanglement in a monochorionic diamniotic twin pregnancy complicated by 2nd-trimester single intrauterine demise. *Fetal Diagnosis and Therapy*.

[7] Suzuki S, Ishikawa G, Sawa R, Yoneyama Y, Otsubo Y, Araki T (1999). Iatrogenic monoamniotic twin gestation with progressive twin-twin transfusion syndrome. *Fetal Diagnosis and Therapy*.

[8] Tamura T, Miura A, Suzuki S (2011). Spontaneous disruption of the dividing membrane in monochorionic diamniotic twin pregnancy. *Fetal Diagnosis and Therapy*.

[9] Gilbert WM, Davis SE, Kaplan C, Pretorius D, Merritt TA, Benirschke K (1991). Morbidity associated with prenatal disruption of the dividing membrane in twin gestations. *Obstetrics and Gynecology*.

[10] Chen SE, Trupin L, Trupin S (1994). Antepartum rupture of diamniotic membranes separating monozygotic twins: a case report. *Journal of Reproductive Medicine for the Obstetrician and Gynecologist*.

[11] De Lia JE, Worthington D (2000). Intrauterine sling with umbilical cord entanglement in diamniotic twins. *Ultrasound in Obstetrics and Gynecology*.

[12] Nasrallah FK, Faden YA (2005). Antepartum rupture of the intertwin-dividing membrane in monochorionic diamniotic twins: a case report and review of the literature. *Prenatal Diagnosis*.

[13] Yoshimura K, Aiko Y, Inagaki H, Nakata M, Hachisuga T (2009). Prenatal spontaneous disruption of the dividing membrane in monochorionic diamniotic twins detected at the time of fetoscopic laser photocoagulation. *Journal of Obstetrics and Gynaecology Research*.

[14] Suzuki S, Kaneko K, Shin S, Araki T (2001). Incidence of intrauterine complications in monoamniotic twin gestation. *Archives of Gynecology and Obstetrics*.

[15] Beasley E, Megerian G, Gerson A, Roberts NS (1999). Monoamniotic twins: case series and proposal for antenatal management. *Obstetrics and Gynecology*.

[16] Roqué H, Gillen-Goldstein J, Funai E, Young BK, Lockwood CJ (2003). Perinatal outcomes in monoamniotic gestations. *Journal of Maternal-Fetal and Neonatal Medicine*.

[17] Tessen JA, Zlatnik FJ (1991). Monoamniotic twins: a retrospective controlled study. *Obstetrics and Gynecology*.

[18] Carr SR, Aronson MP, Coustan DR (1990). Survival rates of monoamniotic twins do not decrease after 30 weeks’ gestation. *American Journal of Obstetrics and Gynecology*.

